# Tumor-Infiltrating Lymphocytes and Tumor-Stroma Ratio on Early-Stage Cervix Carcinoma: Prognostic Value of Two Distinct Morphological Patterns of Microenvironment

**DOI:** 10.7759/cureus.45148

**Published:** 2023-09-13

**Authors:** Ailma Larre, Raquel C Fernandes, Giovana J Gerbasi, Filomena M Carvalho

**Affiliations:** 1 Pathology, Faculdade de Medicina da Universidade de Sao Paulo, Hospital Beneficiencia Portuguesa, Sao Paulo, BRA; 2 Pathology, Laboratorio Bacchi, Sao Paulo, BRA; 3 Obstetrics and Gynecology, Instituto Brasileiro de Controle do Cancer, Hospital Israelita Albert Einstein, Sao Paulo, BRA; 4 Pathology, Faculdade de Medicina da Universidade de Sao Paulo, Sao Paulo, BRA

**Keywords:** microenvironment, prognosis, early-stage, reactive stroma, tumor-stroma ratio, cervical carcinoma

## Abstract

Background

Tumor progression is influenced by the complex network of different cellular elements that make up its microenvironment. Tumor-infiltrating lymphocytes (TILs) and stroma characteristics reflect two faces of the intricate mechanisms involved in the tumor-host interaction and can be easily evaluated by routine histological examination. Their prognostic value could be demonstrated in different tumor tumor types, but they are poorly explored in cervical cancer.

Methodology

In this retrospective study, we analyzed the association of TILs, tumor-stroma ratio (TSR), and pattern of stromal fibroblasts with prognosis and classical clinicopathological variables. We studied 61 patients with early-stage cervical cancer. We reviewed histological type, tumor grade, Silva pattern of invasion for adenocarcinomas, tumor thickness, depth of stromal invasion, lymph vascular space invasion, and lymph node status. The median follow-up was 37.77 months (range 4.77 to 112.37 months).

Results

The TSR did not correlate with any clinicopathological features or disease-free and overall survival. On the other hand, the reactive pattern of stroma composed of larger fibroblasts and less collagenization was associated with the FIGO IB2 stage (p=0.04), larger tumor (p=0.03), and deeper infiltration (p=0.005). There were more recurrences in the group of reactive stroma (33.13% vs. 11.5%), although the difference did not reach statistical significance. Reactive stroma was associated with lower survival free of recurrence (p=0.05) and overall survival (p=0.009). High TILs were associated with squamous cell type (p=0.003), higher tumor grade (p=0.02), and more LVSI (p=0.02). Tumors with higher TILs presented higher free recurrence interval (p=0.06) and overall survival (p=0.03). No association was observed between stroma characteristics and TILs.

Conclusions

Our study suggested that although immune activation and stromal changes are important features of microenvironment remodeling during tumoral progression, they are independent, following distinct carcinogenetic pathways. Pathological assessment of stroma characteristics and TILs adds significant prognostic information and demonstrates how a simple routine laboratory assessment can generate a better understanding of biological phenomena.

## Introduction

Cancer treatment strategies have evolved with medicaments that directly target the neoplastic cells and drugs that interfere with tumor microenvironment (TME) elements, such as immunotherapy and drugs that disrupt and inhibit secretory factors produced in the tumor stroma [1]. TME includes endothelial cells, mesenchymal cells, pro-tumorigenic and anti-tumorigenic immune cells, and secretion products by these cells used in cell signaling pathways that maintain carcinogenesis [2]. The fraction of tumor-infiltrating lymphocytes (TILs) is related to immune activation. On the other hand, stroma composition results from the complex mechanisms that occur in the TME. Both contribute to the biological behavior of tumors [2].

Tumor-stroma ratio (TSR) is one of the parameters supposed to reflex the stroma activity. Its prognostic value was demonstrated in many types of tumors, such as, for example, intrahepatic cholangiocarcinoma [3], breast [4], oral squamous cell carcinoma [5], tongue [6], and colorectal cancer [7,8]. We found two studies with cervical cancer, one with 2018 FIGO stage IIIC squamous cell carcinoma [9] and the other with early-stage (IA2-IIA) [9,10], both demonstrating the prognostic value of stroma proportion. In all these studies, stroma-rich tumors present a worse prognosis, although one cannot deny that the composition of the stroma is dynamic and very heterogeneous.

The different cellular elements of the microenvironment have distinct functions in the processes involved in tumor progression. Morphological evaluation of specimens offers an excellent opportunity to understand the background of complex biological phenomena at the subcellular level.

Considering the simplicity of evaluating TSR, morphological characteristics of stromal fibroblasts, and TILs, we aimed to explore their association with prognosis and classical clinicopathological prognostic parameters in a cohort of early-stage cervical carcinomas.

## Materials and methods

This study was approved by the Scientific Committee of the Department of Pathology of Faculdade de Medicina da Universidade de Sao Paulo, by Instituto Brasileiro de Controle do Cancer - IBCC (CAAE 95671518.3.3001.0072), by the Ethics Committee for Research Projects of the Faculdade de Medicina da Universidade de Sao Paulo (# 2.836.508), and Plataforma Brazil (CAAE 95671518.3.0000.0065). It complies with the ethical precepts proposed by the legislation in force in Brazil R466/2012.

In this retrospective study, we included 61 patients with pathological diagnosis of cervical carcinoma, 2009 FIGO stages IB1-IB2, submitted to radical surgical and pelvic lymphadenectomy in IBCC between 2010 and 2017. We retrieved the following information from the medical records: age at diagnosis, clinical 2009 FIGO stage [11], pathological tumor size (mm), and follow-up.

The same pathologist reviewed all slides (RCMF). The discordances between the revision and the original diagnosis were decided after discussion with the third pathologist (FMC). The following characteristics were analyzed: histological type (squamous cell carcinoma or endocervical adenocarcinoma), tumor grade (1, 2, or 3), Silva pattern of invasion for adenocarcinomas [12], tumor thickness (mm), depth of stromal invasion (mm), lymph vascular space invasion (LVSI) (present vs. absent) and lymph node status (negative vs. positive). The Silva pattern of invasion classifies adenocarcinomas into three categories [12]. Pattern A is characterized by groups of well-demarcated round glands without destructive invasion of stroma or LVSI. Pattern B presents limited destructive stromal invasion in a background of Silva A pattern, with or without LVSI. Pattern C is characterized by diffuse destructive invasion, frequent angular glands, and more complex architecture. Depth of invasion was measured from the base of the epithelium of origin to the deepest point of invasion or tumor thickness in cases with difficulty determining the epithelium of origin. Depth of invasion was analyzed as a continuous variable and categorized according to the third of the total cervical thickness.

TSR, stroma characteristics, and TILs were evaluated by light microscopy independently by two senior pathologists (RCMF and FMC) in hematoxylin-eosin whole histological sections. The discordances were analyzed together for consensus.

**Figure 1:**
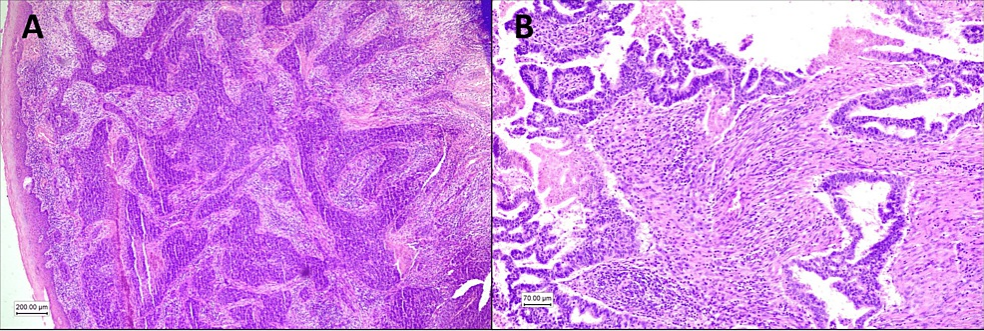
(A) Squamous cell carcinoma with high tumor-stroma ratio (stroma poor). (B) Adenocarcinoma with low tumor-stroma ratio (stroma-rich).

TSR) was assessed in the area with the highest stromal percentage selected with 40x magnification. The chosen area containing both tumor and stroma was examined at 100x magnification. The fields should have tumor cells at the periphery. Tumors with low TSR, with 50% stroma or more in at least one microscope field, were defined as stroma high. Those with less than 50% stroma (high TSR) were defined as stroma-low (Figures 1A, 1B) [10].

**Figure 2:**
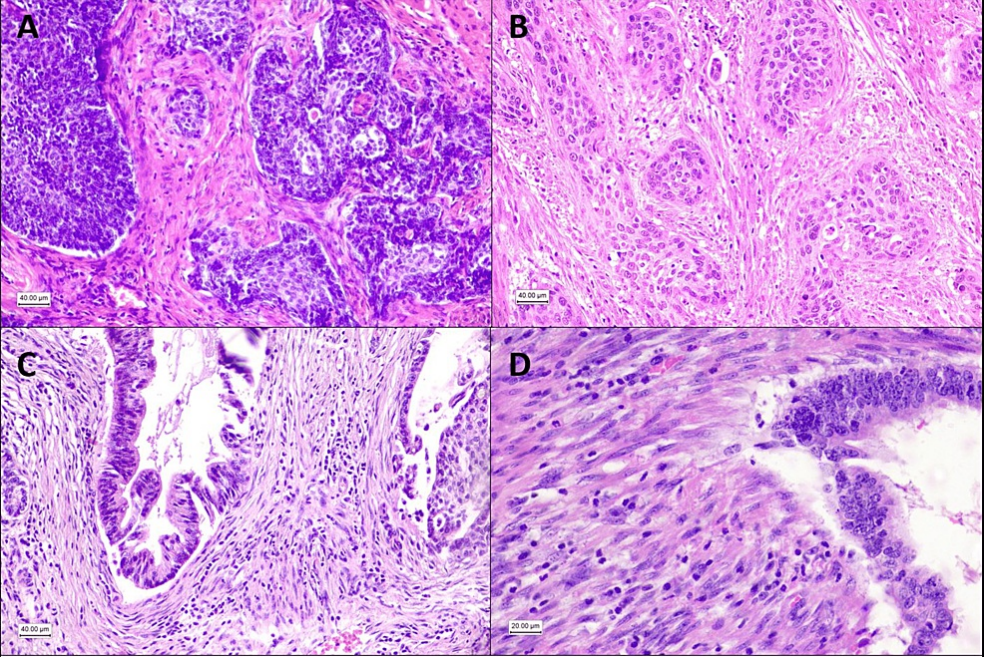
Patterns of stroma fibroblasts in the tumor area. (A) Squamous cell carcinoma with tumoral inactive stroma, composed of normal resident fibroblasts. (B) Squamous cell carcinoma with reactive stroma. (C) Adenocarcinoma with reactive stroma, poor collagenized. (D) Adenocarcinoma with reactive stroma showing spindle fibroblasts with clear nuclei.

Stroma was classified according to the pattern of fibroblasts and their products into reactive or inactive. Reactive stroma was characterized by signs of moderate or intense desmoplasia, with stromal cells of the myofibroblastic type, larger than the normal fibroblast, spindle, or stellate, with fibrillar cytoplasm and often indented nuclei, associated to altered stroma (poorly collagenized, edematous, myxoid or keloid-like) [13]. All the subtypes of desmoplastic reaction described by Ueno et al. [14] were put together (Figures 2A-2D).

**Figure 3:**
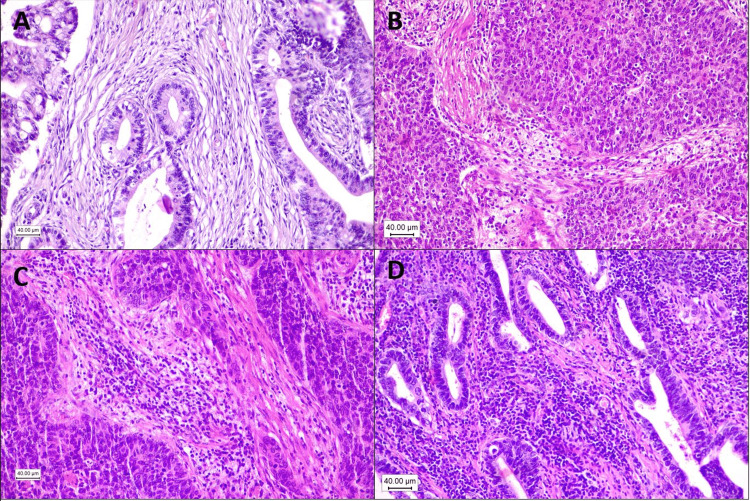
Tumor-infiltrating lymphocytes (TILs). (A) Adenocarcinoma with 5% of TILs (TILs-low). (B) Squamous cell carcinoma with 10% of TILs (TILs-low). (C) Squamous cell carcinoma with 30% of TILs (TILs-high). (D) Adenocarcinoma with 80% of TILs (TILs-high).

Stromal tumor-infiltrating lymphocytes (TILs) were evaluated as the percentage of mononuclear immune cells, including lymphocytes and plasma cells, in the tumor area, without direct contact with tumor cells, according to the criteria of Salgado et al. [15]. The percentage was defined by the area of tumor stroma occupied by the mononuclear immune cells. Although the microscope magnification supposedly makes no difference, we evaluated it at 200x magnification. We arbitrarily defined the median as the cut-off of the subgroups TILs-high (>median) and TILs-low (≤median) (Figures 3A-3D).

The chi-square or Fisher’s exact test evaluated the association between categorical variables. Comparison of the groups according to the continuous variable (age, tumor size, tumor thickness, and depth of invasion) was performed with the Kruskal-Wallis test. Overall survival (OS)was defined by the time from surgery to death, while recurrence-free survival (RFS) as the time from surgery to the time of recurrence or death. Kaplan-Meier curves were applied to describe RFS and OS, calculated over three and five years. The log-rank test compared survival curves. Two-tailed p-values less than 0.05 were considered significant. MedCalc® Software, version 20.218 (BVBA, Ostend, Belgium) conducted all statistical analyses.

## Results

The age of patients ranged from 25 y to 76 y (median 41 (95% confidence interval (CI) 37-43.6), and they were followed up for a median of 37.77 months (range 4.77 to 112.37 months). Clinicopathological characteristics are summarized in Table 1.

**Table 1:** Clinicopathological characteristics of 61 patients with 2009 FIGO stages IB1-IB2 cervical cancer after radical surgery CI: Confidence Interval; FIGO: International Federation of Obstetrics and Gynecology; LVSI: lymph vascular space invasion

Characteristics	n
Age (years)	
Median (95% CI)	41 (37-43.6)
range	25-76
2009 FIGO stage	
IB1	45 (73.8%)
IB2	16 (26.2%)
Histological type	
Squamous cell carcinoma	31 (50.8%)
Adenocarcinoma	30 (49.2%)
Silva system	
A	7 (23.3%)
B	2 (6.7%)
C	21 (70.0%)
Tumor grade	
1	15 (24.6%)
2	25 (41.0%)
3	21 (34.4%)
Tumor size (mm)	
Median (95% CI)	25 (22-30)
range	4-60
Tumor thickness (mm)	
Median (95% CI)	10 (8.2-13)
range	2-35
Depth of infiltration (mm)	
Median (95% CI)	6 (5-9)
range	2-27
Depth of infiltration (thirds)	
Inner	28 (45.9%)
middle	16 (26.2%)
outer	17 (27.9%)
LVSI	
Present	14 (23.0%)
Not identified	47 (77.0%)
Tumor-infiltrating lymphocytes (%)	
Median (95% CI)	20 (10-30)
range	5-100
Lymph node status	
Negative	51 (87.9%)
Positive	7 (12.1%)
Recurrence	
No	52 (85.2%)
yes	9 (14.8%)
Survival status	
alive	56 (91.8%)
Deceased	5 (8.2%)

Comparisons of clinicopathological characteristics according to TSR (stroma-high vs. stroma-low) are presented in Table 2.

**Table 2:** Clinicopathological characteristics of 61 patients with 2009 FIGO stages IB1-IB2 cervical cancer after radical surgery according to tumor-stroma ratio FIGO: International Federation of Obstetrics and Gynecology; LVSI: lymph vascular space invasion; * Kruskal-Wallis; ** Fisher’s exact test; ***Chi-square test

Characteristics	Low tumor-stroma ratio (Stroma-high)	Stroma-low High tumor-stroma ratio (stroma-low)	p
n	10	51	
Age (years)			
Median	44	39	0.085*
range	28-76	25-74	
2009 FIGO stage			
IB1	7 (70%)	38 (74.5%)	0.71**
IB2	3 (30%	13 (25.5%)	
Histological type			
Squamous cell carcinoma	5 (50%)	26 (51%)	1**
Adenocarcinoma	5 (50%)	25 (49%)	
Silva system			
A	0	7	0.28***
B	0	2	
C	5	16	
Tumor grade			
1	2 (20%)	13 (25.5%)	0.52***
2	3 (30%)	22 (43.1%)	
3	5 (50%)	16 (31.4%)	
Tumor size (mm)			
Median	22.5	25	0.50*
range	5-56	4-60	
Tumor thickness (mm)			
Median	9.75	10	0.93*
range	5-25	2-35	
Depth of infiltration (mm)			
Median	6.5	6	0.59*
range	5-18	2-27	
Depth of infiltration (thirds)			
Inner	3 (30%)	25 (49%)	0.46***
middle	4 (40%)	12 (23.5%)	
outer	3 (30%)	14 (27.5%)	
LVSI			
Present	4 (40%)	10 (19.6%)	0.22**
Not identified	6 (60%)	41 (80.4%)	
Tumor-infiltrating lymphocytes (%)			
median	20	20	0.53*
range	5-90	5-100	
Lymph node status			
Negative	7 (77.8%)	44 (89.8%)	0.30**
Positive	2 (22.2%)	5 (10.2%)	
Recurrence			
No	9 (90%)	43 (84.3%)	1**
yes	1 (10%)	8 (15.7%)	
Survival status			
alive	8 (80%)	48 (94.1%)	0.18**
deceased	2 (20%)	3 (5.9%)	

There was no association with any of the clinicopathological features. However, when analyzing the stromal pattern, whether reactive or inactive, we observed an association between reactive stroma and FIGO IB2 stage (p=0.04), larger tumor (p=0.03), and deeper infiltration (p=0.005). No association was observed with histological tumor type, tumor grade, or LVSI, although there were more positive lymph nodes (33.3% vs. 8.2%, non-significant). There were more recurrences in the group of reactive stroma (33.13% vs. 11.5%), although the difference did not reach statistical significance. The number of deaths was higher among tumors with reactive stroma (33.3% vs. 3.8%, p=0.02).

**Figure 4:**
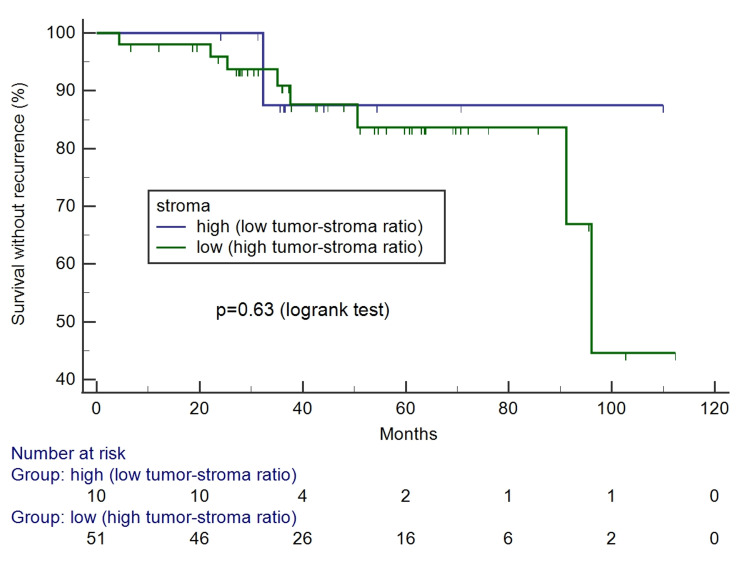
Kaplan-Meier survival curves for free of recurrence interval according to tumor-stroma ratio.

**Figure 5:**
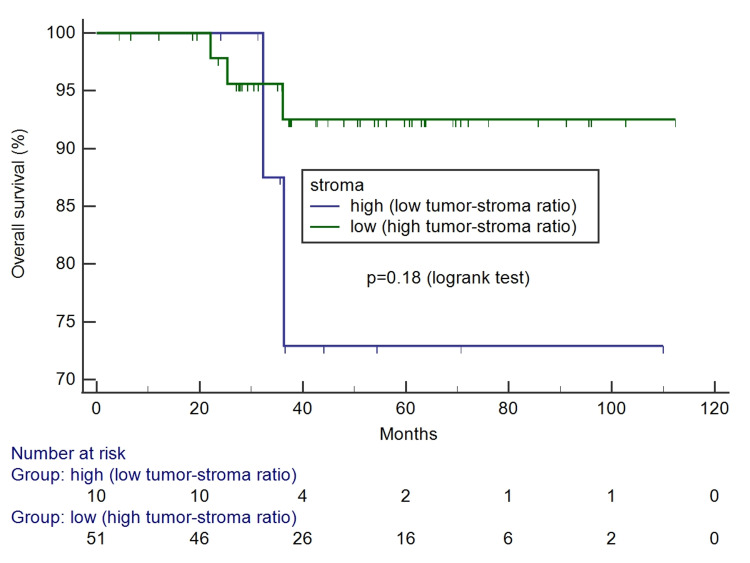
Kaplan-Meier survival curves for overall survival according tumor-stroma ratio.

**Figure 6:**
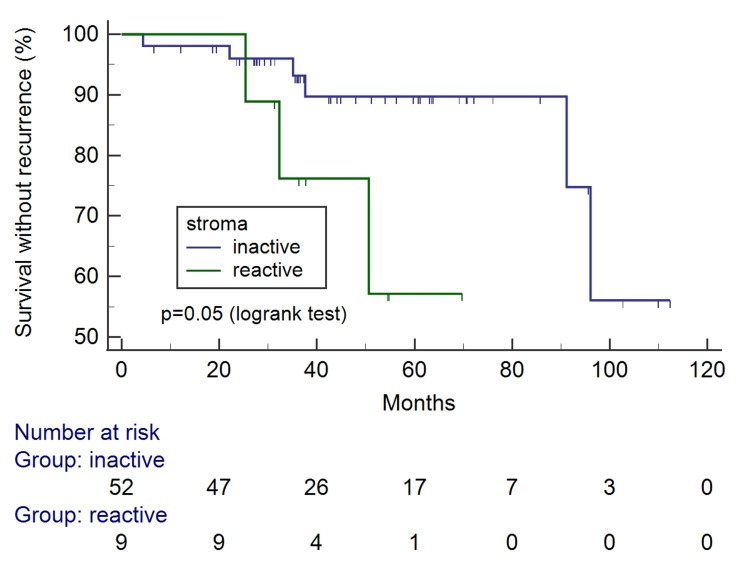
Kaplan-Meier survival curves for free of recurrence interval according to stroma pattern.

**Figure 7:**
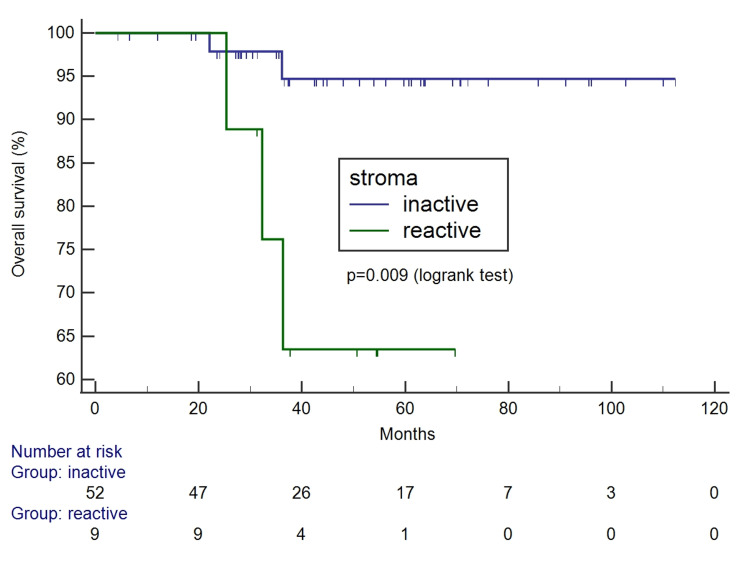
Kaplan-Meier survival curves for overall survival according to according to stroma pattern.

The Kaplan-Meier survival curves according to tumor-stroma ratio and stroma pattern can be seen in Figures 4-7. Only the reactive pattern of the stroma was associated with recurrence and death.

There was an association between TSR with stroma pattern: four (40%) of the 10 cases with stroma-high were of the reactive type, while five (9.8%) of the 51 cases of stroma-low were reactive (p=0.01). Comparisons of clinicopathological characteristics according to the type of fibroblastic component (reactive vs. inactive) are presented in Table 3.

**Table 3:** Clinicopathological characteristics of 61 patients with 2009 FIGO stages IB1-IB2 cervical cancer after radical surgery according to tumoral stroma pattern FIGO: International Federation of Obstetrics and Gynecology; LVSI: lymph vascular space invasion; * Kruskal-Wallis; ** Fisher’s exact test; ***Chi-square test

Characteristics	Inactive stroma	Reactive stroma	p
n	52	9	
Age (years)			
Median	39.5	48	0.08*
range	25-76	32-69	
2009 FIGO stage			
IB1	41 (78.8%)	4 (44.4%)	0.04**
IB2	11 (21.2%)	5 (55.6%)	
Histological type			
Squamous cell carcinoma	27 (51.9%)	4 (44.4%)	0.73**
Adenocarcinoma	25 (48.1%)	5 (55.6%)	
Silva system			
A	7 (28%)	0	
B	2 (8%)	0	
C	16 (64%)	5 (100%)	
Tumor grade			
1	12 (23.1%)	3 (33.3%)	0.79***
2	22 (42.3%)	3 (33.3%)	
3	18 (34.6%)	3 (33.3%)	
Tumor size (mm)			
Median	22	32	0.03*
range	4-60	25-52	
Tumor thickness (mm)			
Median	9.5	16	0.02*
range	2-30	9-35	
Depth of infiltration (mm)			
Median	6	11	0.09*
range	2-27	2-20	
Depth of infiltration (thirds)			
Inner	28 (53.8%)	0	0.005***
middle	13 (25%)	3 (33.3%)	
outer	11 (21.2%)	6 (66.7%)	
LVSI			
Present	11 (21.2%)	3 (33.3%)	0.42**
Not identified	41 (78.8%)	6 (66.7%)	
Tumor-infiltrating lymphocytes (%)			
median	30	10	0.06*
range	5-100	5-50	
Lymph node status			
Negative	45 (91.8%)	6 (66.7%)	0.07**
Positive	4 (8.2%)	3 (33.3%)	
Recurrence			
No	46 (88.5%)	6 (66.7%)	0.12**
yes	6 (11.5%)	3 (33.3%)	
Survival status			
alive	50 (96.2%)	6 (66.7%)	0.02**
deceased	2 (3.8%)	3 (33.3%)	

**Figure 8:**
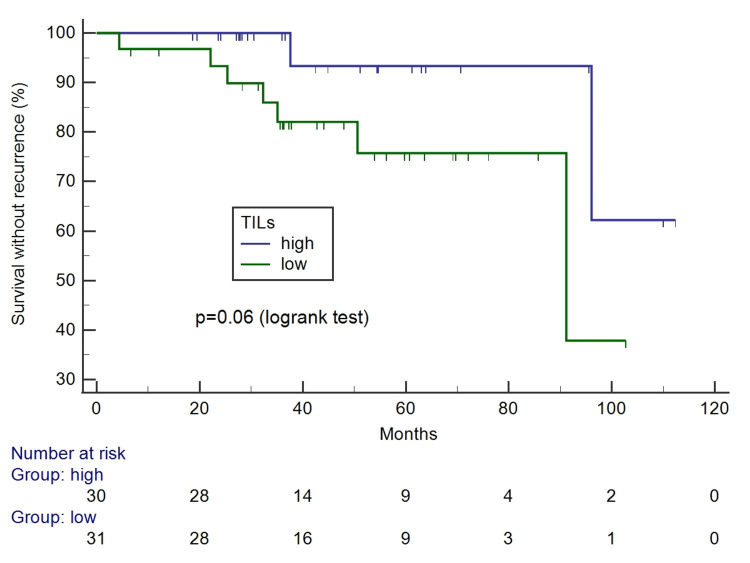
Kaplan-Meier survival curves for free of recurrence interval according to tumor-infiltrating lymphocytes.

**Figure 9:**
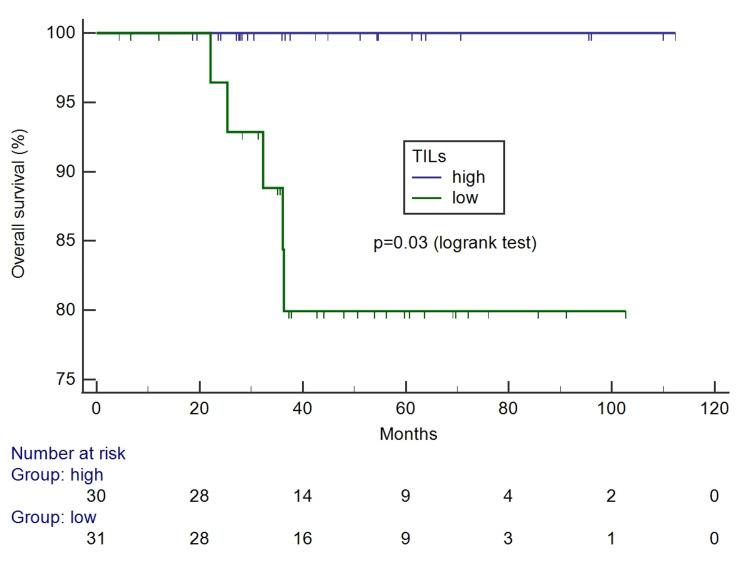
Kaplan-Meier survival curves for overall survival according to tumor-infiltrating lymphocytes.

The percentage of TILs ranged from 5% to 100%, with a mean of 33.03 and a standard deviation of 30.25. The median was 20% and was applied to classify tumors with high-TILs (values above 20%) and low-TILs (up to 20%). The survival curves according to TILs levels are presented in Figures 8, 9.

Comparisons of clinicopathological characteristics according to TILs (high vs. low) are presented in Table 4. High-TILs were associated with squamous cell type (p=0.003), higher tumor grade (p=0.02), and more LVSI (p=0.02). No association was observed with the other characteristics, including lymph node status. All deceased patients had tumors with low levels of TILs. There were more recurrences among low-TILs (22.6% vs. 6.7%), but the difference did not reach the significance level. The adverse outcome was associated with low levels of TILs.

**Table 4:** Clinicopathological characteristics of 61 patients with 2009 FIGO stages IB1-IB2 cervical cancer after radical surgery according to tumor-infiltrating lymphocytes (TILs) FIGO: International Federation of Obstetrics and Gynecology; LVSI: lymph vascular space invasion; * Kruskal-Wallis; ** Fisher’s exact test; ***Chi-square test

Characteristics	TILs high	TILs low	p
n	30	31	
Age (years)			
Median	39.5	42	0.31*
range	25-63	28-76	
2009 FIGO stage			
IB1	25 (83.3%)	20 (44.4%)	0.10**
IB2	5 (16.7%)	11 (35.5%)	
Histological type			
Squamous cell carcinoma	21 (70%)	10 (32.3%)	0.003**
Adenocarcinoma	9 (30%)	21 (67.7%)	
Silva system			
A	0	7 (33.3%)	--
B	2 (22,2%)	0	
C	7 (77.8%)	14 (66.7%)	
Silva system			
A/B	2 (22.2%)	7 (33.3%)	0.68**
C	7 (77.8%)	14 (66.7%)	
Tumor grade			
1	4 (13,3%)	11 (35.5%)	0.02***
2	12 (40.0%)	13 (41.9%)	
3	14 (46.7%)	7 (22.6%)	
Tumor size (mm)			
Median	25	25	0.73*
range	9-60	4-52	
Tumor thickness (mm)			
Median	10	10	0.53*
range	2.5-30	2-35	
Depth of infiltration (mm)			
Median	8.25	5	0.17*
range	2-23	2-27	
Depth of infiltration (thirds)			
Inner	15 (50%)	13 (41.9%)	0.80***
middle	7 (23.3%)	9 (29%)	
outer	8 (26.7%)	9 (29%)	
LVSI			
Present	11 (36.7%)	3 (9.7%)	0.02
Not identified	19 (63.3%)	28 (90.3%)	
Lymph node status			
Negative	25 (86.2%)	26 (89.7%)	1**
Positive	4 (13.8%)	3 (10.3%)	
Recurrence			
No	28 (93.3%)	24 (46.2%)	0.15**
yes	2 (6.7%)	7 (22.6%)	
Survival status			
alive	30 (100%)	26 (83.9%)	0.05**
deceased	0	5 (16.1%)	

After observing the dissociation between LVSI and lymph node status according to stroma pattern and TILs, we checked and confirmed the association between LVSI and lymph node status. Lymph node metastasis was more often among LVSI-positive than negative (30.8% vs. 6,7%, p=0.02). No association was observed between TSR, stroma characteristics, and TILs.

## Discussion

Brazil, as in other low- and low-middle-income countries, has a high incidence of cervical cancer, with 17,010 new cases/year estimated in 2023-2025, representing a risk of 15.38 cases per 100,000 women [16]. Unfortunately, most cases arrive locally advanced or advanced at diagnosis [17]. Here, we set out to explore some characteristics of tumor stroma of early cervical cancers, trying to understand what happens in the first steps of the disease.

Immune and stromal cells are some of the elements capable of expressing the backstage of these phenomena. Immune cells can be divided into two major subgroups: immune suppressors and immune stimulatory cells [18], both components of the TILs population and used as the result of the immune response. Neoantigens expressed by tumor cells are identified and processed by antigen-presenting cells (APCs). In lymphoid tissues, APCs activate T lymphocytes by binding T-cell receptors (TCR) to their cognate antigen presented by APCs by the major histocompatibility complexes (MHC). Activated T-cells CD8+ migrate, kill tumor cells, and generate more neoantigens [19]. Several checkpoints control the immune response, some activating, others inhibiting, to maintain equilibrium in normal conditions [20]. However, inhibitory checkpoints in cancer are responsible for immune escape, enhancing tumor progression. The Programmed Cell Death Protein 1 (PD-1) is one of the immune checkpoint inhibitors expressed on the surface of activated T-cells. It binds to its ligand, Programmed Cell Death Ligand 1 (PD-L1), a transmembrane protein in various cell types, including tumor cells. The connection between PD1 and PD-L1 promotes the reduction of harmful immune responses and immune tolerance, acting as a promoting factor in tumors [21]. The rationale for treatment with immune checkpoint inhibitors anti-PD-1, or anti-PD-L1, is to allow the reactivation of cytotoxic T cells to kill tumor cells. TILs evaluation reflects the immune response and has been proposed as a biomarker for immune checkpoint inhibitors [22].

Other stromal cells, besides the immune cells, have received less attention, particularly when considering pathological evaluation because they have a different role of TILs in the therapeutic decision. However, there were no doubts about the importance of fibroblasts and other cells in the biological behavior of cancers [23]. Fibroblasts are the most important stroma component, especially the cancer-associated fibroblasts (CAFs). Normal fibroblasts are resident cells that initially have anti-tumorigenic activity, while CAFs correspond to activated cells with a pro-tumoral role [24].

Several studies with different types of cancer considered the TSR as a parameter of the activity of stroma and classified the neoplasms as stroma-rich and stroma-poor [4,5,8,25-27]. Dourado et al. and Kang et al., both studies with oral carcinomas, defined stroma-rich tumors as those with ≥50% stroma fraction, with low TSR, the same criteria as ours [5,25]. Others, such as Xu et al. with urothelial carcinomas, and Dang et al. and Smit et al. with colorectal cancer, applied the criteria of >50% of stroma fraction to define the stroma-rich group [8,26,27]. Yan et al., in turn, used a different cut-off (33.5%), although, like most of the studies, the authors reported a poorer prognosis among patients with stroma-rich breast cancer [4]. In these studies, a higher area of the stroma than the tumor area was associated with a poorer prognosis, except Dang et al. did not demonstrate an increased risk for an adverse outcome in non-pedunculated T1 colorectal cancer [27], although in other stages the prognostic impact of TSR is well demonstrated [7,8]. Few studies evaluated cervical cancer. Zong et al. studied 384 patients with 2018 FIGO stage IIIC cervical cancer and found an association between stroma-rich, defined by ≥50% of stroma fraction, with poorer survival [9,10]. Liu et al. presented a cohort of 184 patients with early-stage cervical cancer, and they also found a worse prognosis in the stroma-rich group [10]. Although our sample was small, with only nine recurrences and five deaths, our results differed. We did not observe any difference between high- and low TSR. Observing the figures of histological sections in the articles mentioned above, we noticed that the stroma-rich was illustrated by an intense desmoplasia composed of active fibroblasts and not the normal resident cells. So, we decided to analyze the type of stroma (active vs. inactive) instead of the TSR, and we found similar results to theirs, i.e., poorer prognosis in the group of active stroma. Besides, stroma with active fibroblasts was associated with larger tumors and deeper infiltration. A possible reason for this finding is that in early cervical cancer, the cervical stroma predominantly comprises resident cells, i.e., fibroblasts that initially have anti-tumorigenic activity. With the local progression of the disease (larger tumors, more infiltrative), the stroma becomes reactive, with fibroblasts with pro-tumoral activity.

The association of TILs with prognosis in cervical cancer has been well documented [28]. Stromal TILs (sTILs), i.e., the proportion of lymphocytes in areas between carcinoma cells that are not in direct contact with tumor cells, are easier to evaluate than intratumoral TILs (iTILs). Besides, according to Gultekin et al., sTILs have a superior prognostic impact than iTILs [28]. These authors, like us, defined an arbitrary cut-off for sTILs corresponding to the median. However, this value was higher than in our cases (30% instead of 20%), probably because ours were in earlier stages. Higher TILs values in our cases were associated with squamous histological type and high tumor grade. High-grade tumors are less differentiated and have, in general, more neoantigens, so they are expected to elicit greater immune activation. Although more LVSI was identified among TILs-high tumors, we did not observe any difference in lymph node status. Results were identical to the study done by Ohno et al. [29]. IB1 2009 FIGO stage was more frequent than IB2 among the TILs-high group, but when we compared the tumor size, tumor thickness, or depth of infiltration, there was no difference between the two groups. To explain these findings, we can suppose that local tumor growth, at least in the early stages, is not influenced by the immune system. All the deaths occurred in patients with tumors with TILs-low. Several studies, some exploring subtypes of TILs, obtained similar results [30-32]. Although not for cervical cancer, an immunoscore was validated for colon cancer, determined by the digital analyses of densities of CD3+ and CD8+ T cells in tumor and invasive margin [33]. Immunoscore association with recurrence was independent of patient age, sex, American Joint Committee on Cancer and Union for International Cancer Control TNM staging system, microsatellite instability, and classical prognostic factors in colon cancer. An interesting finding was that of Ohno et al., who also studied different types of TILs, including Treg cells, supposedly immunosuppressive, and found higher values of all the cell types associated with better prognosis [29]. We speculate that tumor progression may be associated with developing immune escape mechanisms or intrinsic tumor biological properties related to low immune activation. Wang et al. evaluated the changes in the immune infiltration from normal to invasive carcinoma, passing through precursor lesions. They observed the triggering of immunosuppression mechanisms at precancerous stages, an increase of innate and adoptive cells from normalcy to cancer, a decrease of cytotoxic T-cells with the development of cancer, and immune escape mechanisms increasing from high-grade squamous intraepithelial lesion to cancer [33]. Our results suggest that TILs reflect immune activation secondary to the intrinsic properties of neoplastic cells. On the other hand, activated fibroblasts reflect a more complex tumor phenotype due to interaction with the microenvironment.

This study has strengths and weaknesses. Although small to really corroborate with survival, the cohort is relatively homogeneous regarding the stage of the disease (only stage IB) and treatment, as all cases were treated by the same team at the same Institution. Our encouraging results show an opportunity for better exploration of histological characteristics as indicators of complex biological processes.

## Conclusions

Our study demonstrated that TILs and the type of fibroblasts in the tumoral stroma are implicated in the prognosis of cervical cancer. Inactive stroma, composed of normal, resident fibroblasts, and high values of TILS are associated with better prognosis. Reactive stroma is associated with a larger volume of tumor and deeper infiltration, while high TILs were more frequent among squamous cell carcinoma, higher tumor grade, and more LVSI. The results suggest immune activation and stromal changes during tumoral progression develop through different pathways. These easy histopathological assessments should be considered standard routine in diagnostic work-up.

## References

[1] Xu M, Zhang T, Xia R, Wei Y, Wei X (2022). Targeting the tumor stroma for cancer therapy. Mol Cancer.

[2] Hanahan D (2022). Hallmarks of cancer: new dimensions. Cancer Discov.

[3] Guedj N, Blaise L, Cauchy F, Albuquerque M, Soubrane O, Paradis V (2021). Prognostic value of desmoplastic stroma in intrahepatic cholangiocarcinoma. Mod Pathol.

[4] Yan D, Ju X, Luo B, Guan F, He H, Yan H, Yuan J (2022). Tumour stroma ratio is a potential predictor for 5-year disease-free survival in breast cancer. BMC Cancer.

[5] Dourado MR, Miwa KY, Hamada GB (2020). Prognostication for oral squamous cell carcinoma patients based on the tumour-stroma ratio and tumour budding. Histopathology.

[6] Sakai T, Saito Y, Tateishi Y (2022). Tumor-stroma ratio can predict lymph-node metastasis in cT1/2N0 oral tongue squamous cell carcinoma independent of tumor budding grade. Int J Clin Oncol.

[7] Sullivan L, Pacheco RR, Kmeid M, Chen A, Lee H (2022). Tumor stroma ratio and its significance in locally advanced colorectal cancer. Curr Oncol.

[8] Smit MA, van Pelt GW, Terpstra V, Putter H, Tollenaar RA, Mesker WE, van Krieken JH (2021). Tumour-stroma ratio outperforms tumour budding as biomarker in colon cancer: a cohort study. Int J Colorectal Dis.

[9] Zong L, Zhang Q, Kong Y (2020). The tumor-stroma ratio is an independent predictor of survival in patients with 2018 FIGO stage IIIC squamous cell carcinoma of the cervix following primary radical surgery. Gynecol Oncol.

[10] Liu J, Liu J, Li J (2014). Tumor-stroma ratio is an independent predictor for survival in early cervical carcinoma. Gynecol Oncol.

[11] Pecorelli S, Zigliani L, Odicino F (2009). Revised FIGO staging for carcinoma of the cervix. Int J Gynaecol Obstet.

[12] Diaz De Vivar A, Roma AA, Park KJ (2013). Invasive endocervical adenocarcinoma: proposal for a new pattern-based classification system with significant clinical implications: a multi-institutional study. Int J Gynecol Pathol.

[13] Mills SE (2019). Histology for pathologists. LWW.

[14] Ueno H, Konishi T, Ishikawa Y (2014). Histologic categorization of fibrotic cancer stroma in the primary tumor is an independent prognostic index in resectable colorectal liver metastasis. Am J Surg Pathol.

[15] Salgado R, Denkert C, Demaria S (2015). The evaluation of tumor-infiltrating lymphocytes (TILs) in breast cancer: recommendations by an International TILs Working Group 2014. Ann Oncol.

[16] Estimativa 2023 (2023). Incidência de Câncer no Brasil. (2022). Accessed: 21/04. https://www.inca.gov.br/sites/ufu.sti.inca.local/files//media/document//estimativa-2023.pdf..

[17] Paulino E, de Melo AC, Silva-Filho AL, Maciel LF, Thuler LC, Goss P, Nogueira-Rodrigues A (2020). Panorama of Gynecologic Cancer in Brazil. JCO Glob Oncol.

[18] Kousar K, Ahmad T, Naseer F, Kakar S, Anjum S (2022). Review Article: Immune Landscape and Immunotherapy Options in Cervical Carcinoma. Cancers (Basel).

[19] Kumar V, Abbas AK, Aster JC (2020). Robbins and Cotran Pathologic Basis of Disease. In Robbins Pathology. Tenth edition. edition.

[20] Schütz F, Stefanovic S, Mayer L, von Au A, Domschke C, Sohn C (2017). PD-1/PD-L1 Pathway in Breast Cancer. Oncol Res Treat.

[21] Han Y, Liu D, Li L (2020). PD-1/PD-L1 pathway: current researches in cancer. Am J Cancer Res.

[22] El Bairi K, Haynes HR, Blackley E (2021). The tale of TILs in breast cancer: A report from The International Immuno-Oncology Biomarker Working Group. NPJ Breast Cancer.

[23] Dzobo K, Senthebane DA, Dandara C (2023). The Tumor Microenvironment in Tumorigenesis and Therapy Resistance Revisited. Cancers (Basel).

[24] Kang J, Su M, Xu Q, Wang C, Yuan X, Han Z (2023). Tumour-stroma ratio is a valuable prognostic factor for oral tongue squamous cell carcinoma. Oral Dis.

[25] Xu L, Zhong W, Li C (2023). The tumour-associated stroma correlates with poor clinical outcomes and immunoevasive contexture in patients with upper tract urothelial carcinoma: results from a multicenter real-world study (TSU-01 Study). Br J Cancer.

[26] Dang H, van Pelt GW, Haasnoot KJ (2021). Tumour‐stroma ratio has poor prognostic value in nonpedunculated T1 colorectal cancer: A multicentre case‐cohort study. United European Gastroenterol J.

[27] Santoro A, Inzani F, Angelico G (2023). Recent Advances in Cervical Cancer Management: A Review on Novel Prognostic Factors in Primary and Recurrent Tumors. Cancers (Basel).

[28] Gultekin M, Beduk Esen CS, Ates Ozdemir D, Yildirim S, Yuce D, Usubutun A, Yildiz F (2023). Stromal or intraepithelial tumor-infiltrating lymphocytes: which one has more prognostic significance in cervical cancer?. Arch Gynecol Obstet.

[29] Ohno A, Iwata T, Katoh Y (2020). Tumor-infiltrating lymphocytes predict survival outcomes in patients with cervical cancer treated with concurrent chemoradiotherapy. Gynecol Oncol.

[30] Nedergaard BS, Ladekarl M, Nyengaard JR, Nielsen K (2008). A comparative study of the cellular immune response in patients with stage IB cervical squamous cell carcinoma. Low numbers of several immune cell subtypes are strongly associated with relapse of disease within 5 years. Gynecol Oncol.

[31] Cao L, Sun PL, He Y, Yao M, Gao H (2020). Immune stromal features in cervical squamous cell carcinoma are prognostic factors for distant metastasis: A retrospective study. Pathol Res Pract.

[32] Pagès F, Mlecnik B, Marliot F (2018). International validation of the consensus Immunoscore for the classification of colon cancer: a prognostic and accuracy study. Lancet.

[33] Wang Y, He M, Zhang G, Cao K, Yang M, Zhang H, Liu H (2021). The immune landscape during the tumorigenesis of cervical cancer. Cancer Med.

